# CT-guided bone biopsies with non-diagnostic results in pediatric patients—a multi-institutional 10-year retrospective review

**DOI:** 10.1007/s00330-025-12128-5

**Published:** 2025-11-08

**Authors:** Pak Lun Lam, Kin Fen Kevin Fung, Stefanie Wai Ying Yip, Pui Kwan Joyce Chan, Fong Ying Fiona Wan, Amanda Nim Chi Kan, Yee Ling Elaine Kan, Kwok Chuen Wong, Kwok Chung Lai, James Francis Griffith

**Affiliations:** 1 Department of Diagnostic and Interventional Radiology, Kwong Wah Hospital, Kowloon, Hong Kong SAR; 2 Department of Radiology, Hong Kong Children’s Hospital, Kowloon, Hong Kong SAR; 3 Department of Imaging and Interventional Radiology, Prince of Wales Hospital, Shatin, Hong Kong SAR; 4 Department of Diagnostic and Interventional Radiology, Queen Elizabeth Hospital, Kowloon, Hong Kong SAR; 5 Department of Pathology, Hong Kong Children’s Hospital, Kowloon, Hong Kong SAR; 6 Department of Orthopaedics and Traumatology, Prince of Wales Hospital, Shatin, Hong Kong SAR; 7 Department of Imaging and Interventional Radiology, Faculty of Medicine, The Chinese University of Hong Kong, Shattin, Hong Kong SAR

**Keywords:** Bone, Computed tomography, Children, Biopsy (image-guided), X-ray

## Abstract

**Objective:**

This study aimed to determine the diagnostic yield of CT-guided bone biopsies in pediatric patients, the outcome of non-diagnostic CT biopsy results, and to establish factors associated with non-diagnostic biopsy results.

**Materials and methods:**

This is a retrospective study of consecutive pediatric patients ≤ 21 years who underwent CT-guided bone biopsies in three tertiary referral hospitals from December 2011 to March 2022. Clinical information, pre-biopsy CT and MRI images, procedural details, pathological results, and follow-up were assessed. Fisher’s exact test was used to compare categorical variables. Mann–Whitney *U*-test and unpaired *t*-test were used to compare non-parametric and parametric variables, respectively. Statistical significance was set a *p* < 0.05.

**Results:**

A total of 138 patients (mean age 13.9 ± 4.5 years; 95 (60%) male patients) with 157 CT-guided bone biopsies were studied, which yielded 38.2% (60/157) non-diagnostic, 23.6% (37/157) benign, and 38.2% (60/157) malignant results. Most non-diagnostic lesions (88.3% [53/60]) were subsequently determined to be benign. Factors associated with non-diagnostic biopsy results were cystic lesions (*p* = 0.003) incidental lesions (*p* = 0.03), fewer (*p* = 0.02) and shorter (*p* = 0.01) tissue cores, non-aggressive radiological features, including narrow zone of transition (*p* < 0.001), sclerotic margin (*p* < 0.001), no cortical destruction (*p* < 0.001), no periosteal reaction (*p* < 0.001), or no extra-osseous soft tissue mass (*p* < 0.001).

**Conclusion:**

About one-third of CT-guided bone biopsies in pediatric patients yielded non-diagnostic results, though most were ultimately confirmed to be benign. In children and adolescents with suspected primary bone tumors, CT-guided bone biopsy with non-diagnostic histopathological results strongly favors benignity in lesions with non-aggressive imaging features and should align management towards a more conservative approach.

**Key Points:**

***Question*** *Limited data exist on the prevalence and outcome of non-diagnostic CT-guided bone biopsy in pediatric patients to guide clinical management*.

***Findings*** *About one-third of CT-guided bone biopsies in pediatric patients were non-diagnostic, though most were ultimately benign. Lesions with non-aggressive features were associated with non-diagnostic results*.

***Clinical relevance*** *In the setting of multidisciplinary care for patients with suspected primary bone tumors, non-diagnostic CT-guided bone biopsy—particularly in lesions with non-aggressive imaging features—strongly favors benignity, which should steer management towards a more conservative approach*.

**Graphical Abstract:**

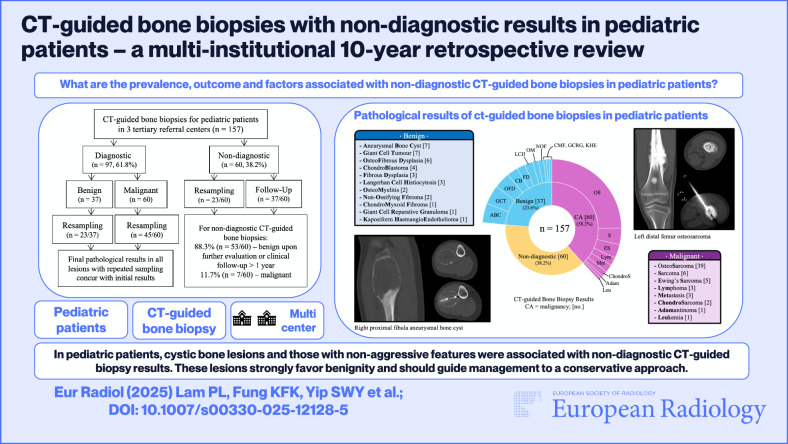

## Introduction

Bone lesions comprise a long list of abnormalities, with a substantial proportion being bone tumors. Typically, they are first assessed radiologically, and often further investigated with CT or MRI [1–3]. Pathological confirmation of bone lesions is necessary when the imaging appearances are not diagnostic, particularly when aggressive features exist [4].

Tissue for histology can be obtained by image-guided biopsy, open biopsy, or surgical resection [5, 6]. CT-guided biopsy is the preferred technique as it is least invasive with low complication risk [7, 8]. In 10–30% of adult cases, image-guided biopsy can yield non-diagnostic histopathological results [9–11]. Further workup of these lesions in the adult population ultimately revealed that nearly half were malignant [10], which is not unexpected, as metastasis is the most common bone tumor in middle-aged adults and the elderly [12].

In pediatric patients, the spectrum of bone lesions is different from adults [13]. Benign primary bone tumors are much more common in children and adolescents than in adults. However, relatively few studies have investigated the prevalence and outcome of lesions with non-diagnostic histopathological results following CT-guided bone biopsies in the pediatric population [14–17]. This study aimed to determine the diagnostic yield of CT-guided bone biopsies in pediatric patients, and to investigate factors associated with non-diagnostic biopsy results.

## Materials and methods

### Study design and patient selection

This retrospective study was approved by the local institutional review board (reference number: PAED-2025-005 (H)). All patients were treated in accordance with the Declaration of Helsinki. The requirement for informed consent was waived.

**Fig. 1:**
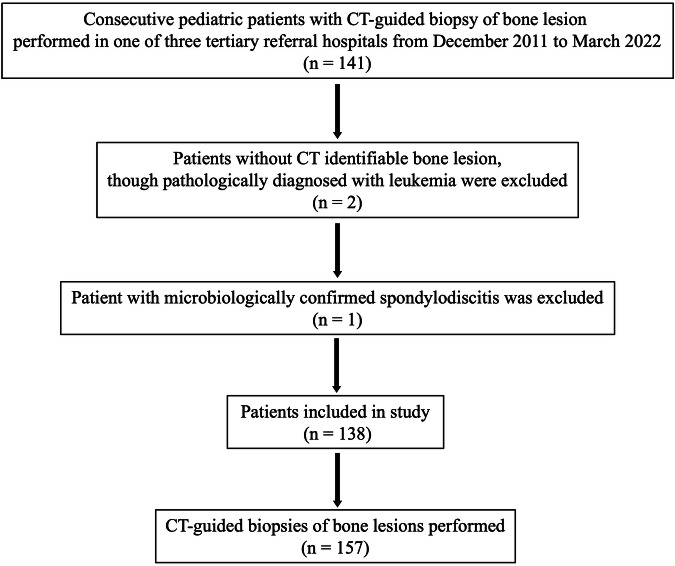
Flow diagram of the inclusion and exclusion process of pediatric patients with CT-guided bone biopsies

The electronic databases of three tertiary referral hospitals for pediatric bone tumor biopsies in the period December 2011 to March 2022 were assessed. Inclusion criteria were pediatric patients aged 21 years or under who underwent CT-guided biopsy for a bone lesion. Exclusion criteria were bone lesions with a known histopathological diagnosis prior to biopsy, bone lesions not identifiable on CT, and spondylodiscitis (Fig. 1). Clinical information, which included age, sex, any known malignancy, and whether the bone lesion was incidentally discovered, or presented with symptoms, was documented.

### Radiological evaluation

Prior to CT-guided biopsy, non-contrast CT (SOMATOM Force, Siemens; SOMATOM Sensation 64, Siemens; Revolution, GE Healthcare; Lightspeed 64 VCT, GE Healthcare) of the targeted bone lesion was performed for pre-procedural planning. CT images were assessed in both bone (level, 700 HU; width, 3000 HU) and soft tissue (level, 40 HU; width, 500 HU) windows, with 0.6 mm orthogonal reformations acquired. All bone lesions were evaluated for location, number, size (maximum diameter on axial, coronal, or sagittal plane), density (lytic, sclerotic, or mixed lytic and sclerotic), zone of transition (narrow or wide), as well as the presence or absence of sclerotic margin, cortical destruction, periosteal reaction, and extra-osseous soft tissue mass.

**Fig. 2:**
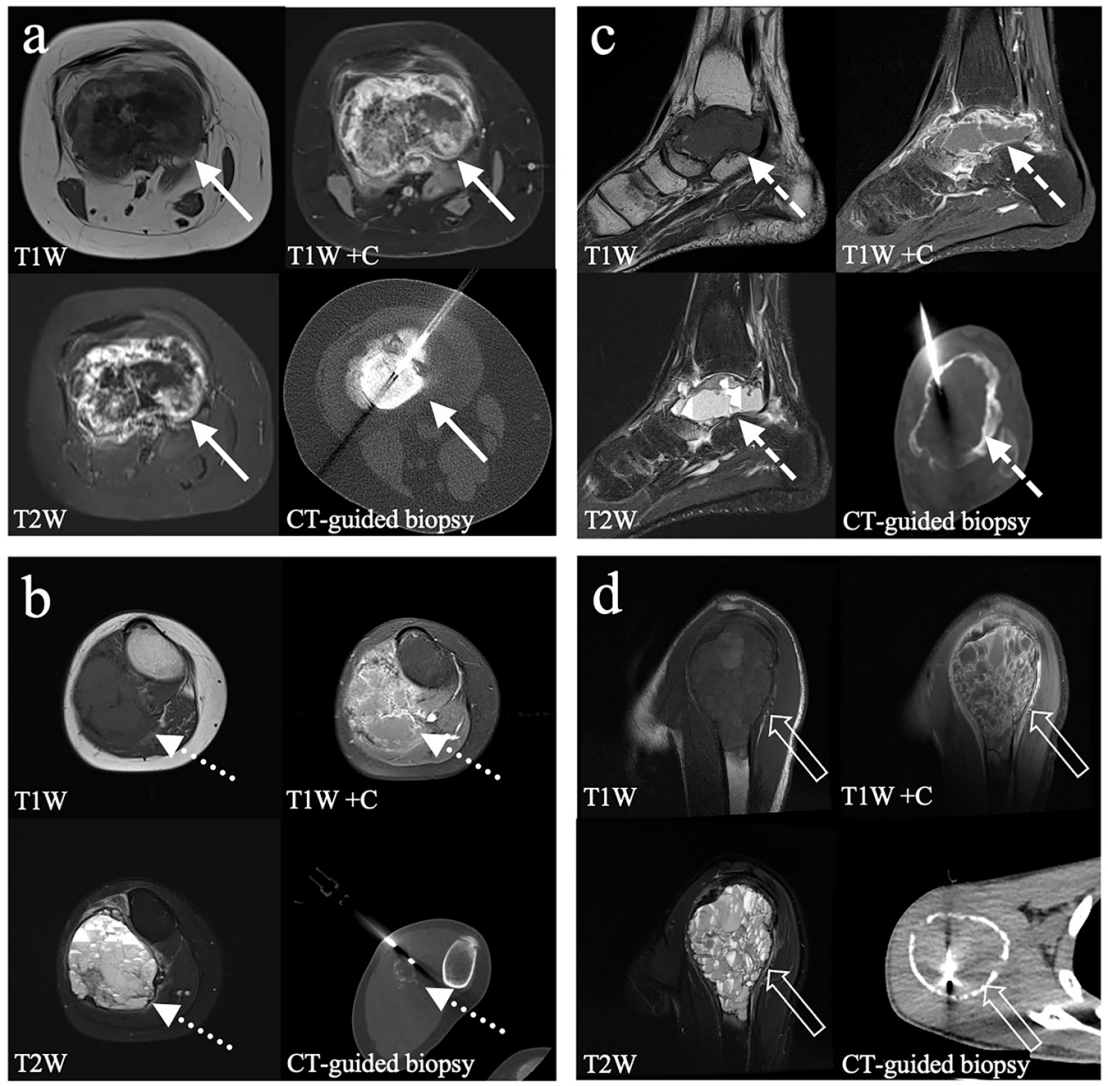
Bone lesions with pre-biopsy contrast-enhanced MRI were categorized according to their proportion of solid and cystic content. **a** Solid lesion (> 90% solid component) distal femur—final diagnosis: osteosarcoma (solid arrows). **b** Predominantly solid lesion (50–90% solid component) proximal fibula—final diagnosis: ewing sarcoma (dotted arrows). **c** Predominantly cystic lesion (> 50–90% cystic component) talus—final diagnosis: chondroblastoma (dashed arrows). **d** Cystic lesion (> 90% cystic component) proximal humerus—final diagnosis: aneurysmal bone cyst (void arrows)

In patients with pre-biopsy MRI (MAGNETOM Aera; MAGNETOM Avanto Fit, Siemens; Achieva X-series, Phillips; MAGNETOM Sonata, Siemens) of the targeted bone lesion, T1-weighted (T1W) images, contrast-enhanced T1W images with fat suppression, and T2-weighted (T2W) images with fat suppression were assessed for cystic content. The cystic versus solid nature of each lesion was assessed based on the relative proportion of solid and cystic components on MRI in a subjective manner. Solid components were defined as areas of solid-appearing tissue usually with enhancement on contrast-enhanced T1W images, while cystic components were identified as fluid-like non-enhancing areas, which typically appeared as low signal on T1W and high signal on T2W sequences. In cases of intralesional hemorrhage, cystic components could demonstrate high T1W and variable T2W signal intensity. Lesions were categorized into four groups: (a) “solid” (> 90% solid component), (b) “predominantly solid” (50–90% solid component), (c) “predominantly cystic” (50–90% cystic component), and (d) “cystic” (> 90% cystic component). Examples are shown in Fig. 2.

The CT and MRI studies were independently reviewed by two radiologists (Reader A—5 years of experience, with subspecialty training in musculoskeletal radiology; Reader B—13 years of experience in pediatric radiology) using a picture archiving and communication system (PACS) workstation. They were blinded to all clinical information. Their evaluations were compared to determine interobserver agreement. All discrepancies were discussed by the two readers and resolved by consensus review. In cases where the consensus could not be reached, adjudication was provided by a third radiologist with more than 30 years of experience in musculoskeletal radiology.

### CT-guided biopsy

The decision to proceed with biopsy was made through multidisciplinary consensus and discussion with parents and/or patients. CT-guided biopsies were performed by ten radiologists, all of whom had at least five years of experience in interventional musculoskeletal radiology. All procedures were performed under sedation or general anesthesia. Local anesthesia (1% lignocaine) was applied to the skin and periosteum, with a maximum dose of 4 mg/kg. The type of bone biopsy needle used was at the discretion of the operator, and included Arrow OnControl Powered Bone Access System (Teleflex), Bonopty biopsy needle (AprioMed), Madison Bone Biopsy System (Merit Medical), Osteobell biopsy needle (Biopsybell Medical), Temno biopsy needle (Merit Medical), and Westbrook Bone Biopsy System (Merit Medical). All biopsy trajectories were formulated in conjunction with pediatric orthopedic surgeons specialized in bone tumor management. For an epiphyseal lesion, the biopsy trajectory was planned to maintain a minimum 1 cm margin from the adjacent growth plate. The needle size, number of biopsy passes, number of tissue cores obtained, total length of the tissue cores, additional biopsy with forceps, type of anesthesia, and any biopsy-related complications were recorded.

### Histopathological examination

Biopsy tissue samples were evaluated by seven pathologists with at least seven years of experience in diagnosing bone and soft tissue tumors. Histopathological assessment, molecular analysis, and cytogenetics, such as fluorescence in situ hybridization and genome sequencing, were performed. Biopsies were defined as diagnostic if a specific benign or malignant diagnosis could be made based on the histological findings. When no specific diagnosis could be established, the biopsy was considered non-diagnostic. The pathological results were further classified into morphological description, no malignancy detected, or insufficient tissue sample.

### Post-biopsy assessment

Any additional histopathological examinations of the bone lesion after initial CT-guided biopsy, including a repeat CT-guided biopsy, open biopsy, surgical resection, and curettage, were documented. Histopathological diagnosis from the final tissue sampling was defined as the reference standard. If a non-diagnostic bone lesion was stable in size or smaller, with alleviation of clinical symptoms on at least one-year follow-up, the lesion was considered benign clinically.

### Statistical analysis

Clinical features, CT and MR features, and procedural details of the CT-guided biopsies were compared between non-diagnostic and diagnostic biopsy groups. Fisher’s exact test was used for categorical data, Mann–Whitney *U*-test was used for non-Gaussian continuous data, and the unpaired *t*-test was used for Gaussian continuous data. Statistical analyses were calculated using SPSS Statistics version 29.0.1.0 (IMB). A *p*-value of < 0.05 was considered statistically significant.

## Results

### Demographics and clinical characteristics

There were 138 patients with 157 CT-guided bone biopsies in this study after exclusion of three patients (biopsy performed for leukemia without CT-identifiable lesion, *n* = 2; microbiologically confirmed spondylodiscitis, *n* = 1) (Fig. 1). Mean age at biopsy was 13.9 ± 4.5 years. There were 95 biopsies (60.5%) performed for male patients, and 62 (39.5%) for female patients. Most lesions (*n* = 144/157, 91.7%) were symptomatic, mainly presenting with pain (Table 1). About two-thirds of the lesions (*n* = 101/157, 64.3%) were in the lower limbs. No complication was encountered in any of the biopsies. Mean duration of clinical follow-up after CT-guided bone biopsy was 4.2 ± 3 years. For non-diagnostic biopsy results, all lesions had interval clinical and radiological follow-up of at least one year. CT-guided bone biopsy details are shown in Tables 2 and 3. For lesions with known malignancy prior to biopsy (14/157, 9.0%), 50% (7/14) showed concordant pathologies with known primary tumor (osteosarcoma = 4, high-grade sarcoma = 1, glioma = 1, and neuroblastoma = 1). The biopsy results for the rest of the lesions (7/14, 50%) were found to be non-diagnostic. Five of the lesions (primary tumor—osteosarcoma = 4; high-grade sarcoma = 1) were considered benign due to clinical or radiological resolution upon follow-up. Two of the lesions (primary tumor—high-grade sarcoma = 1; breast cancer = 1) were considered malignant due to radiological progression.

**Table 1:** Demographics and clinical characteristics of lesions that underwent CT-guided bone biopsies (*n* = 157)

Age (year), mean ± SD	13.9 ± 4.5
Patients aged less than 18 years, no. (%)	131 (83.4)
Patients aged 18–21 years, no. (%)	26 (16.6)
Sex, no. (%)	
Male	95 (60.5)
Female	62 (39.5)
Known malignancy, no. (%)	14 (8.9)
Osteosarcoma	8 (5.1)
High-grade sarcoma*	3 (1.9)
Breast cancer	1 (0.6)
Glioma	1 (0.6)
Neuroblastoma	1 (0.6)
Symptomatic, no. (%)	144 (91.7)
Multiplicity of lesions, no. (%)	132 (84.1)
Monostotic	25 (15.9)
Polyostotic	
Maximum lesion diameter (cm), mean ± SD	6.2 ± 4.0
Follow-up duration after CT-guided bone biopsy (years), mean ± SD (range)	4.2 ± 3.0 (1.0–10.0)

*SD* standard deviation, *no.* number

* Further pathological subtyping was not possible based on the biopsy sample

**Table 2:** Characteristics of CT-guided bone biopsies (*n* = 157)

Characteristics of CT-guided bone biopsies (*n* = 157)	
Bone lesion by anatomical site, no. (%)	
Femur	50 (31.8)
Tibia	42 (26.8)
Humerus	21 (13.4)
Ilium	11 (7.0)
Vertebra	11 (7.0)
Fibula	5 (3.2)
Sacrum	3 (1.9)
Ulna	3 (1.9)
Finger phalanx	2 (1.3)
Metatarsal	2 (1.3)
Radius	2 (1.3)
Rib	2 (1.3)
Calcaneum	1 (0.6)
Pubic ramus	1 (0.6)
Talus	1 (0.6)
Location of lesions within the appendicular bone (*n* = 129), no.	
Diaphysis	28
Metaphysis	92
Epiphysis	9
CT-guided bone biopsies performed by each hospital	
Tertiary referral hospital 1, no. (%)	17 (10.8)
Tertiary referral hospital 2, no. (%)	60 (38.2)
Tertiary referral hospital 1 3, no. (%)	80 (51.0)
Anesthesia, no. (%)	
Moderate sedation	140 (89.2)
Monitored anesthetic care or general anesthesia	17 (10.8)
Biopsy system/needle size (gauge), median (range)	
Outer coaxial system	13.5 (8–17)
Inner biopsy needle	16 (13–18)
No. of biopsy passes, median (range)	4 (1–11)
No. of tissue cores obtained, median (range)	2 (1–10)
Length of tissue cores (cm), mean ± SD	1.3 ± 1.1
Additional biopsy with forceps, no. (%)	25 (15.9)
Complications, no. (%)	0 (0)

*no.* number, *SD* standard deviation

**Table 3:** Histopathological results of CT-guided bone biopsies (*n* = 157)

Histopathological results of CT-guided bone biopsies (*n* = 157)	
Diagnosis	No. of lesions
Specific malignant diagnosis, *n* = 60 (38.2%)	
Osteosarcoma	39
High-grade sarcoma*	6
Ewing’s sarcoma	5
Lymphoma	3
Metastasis	3
Chondrosarcoma	2
Adamantinoma	1
Leukemia	1
Specific benign diagnosis, *n* = 37 (23.6%)	
Aneurysmal bone cyst	7
Giant cell tumor	7
Osteofibrous dysplasia	6
Chondroblastoma	4
Fibrous dysplasia	3
Langerhans cell histiocytosis	3
Osteomyelitis	2
Non-ossifying fibroma	2
Chondromyxoid fibroma	1
Giant cell reparative granuloma	1
Kaposiform haemangioendothelioma	1
Non-diagnostic, *n* = 60 (38.2%)	
Morphological description	37
No malignancy detected	15
Insufficient tissue sample	8

*no.* number

* Further pathological subtyping was not possible based on the biopsy sample

### CT-guided bone biopsies with positive results

**Fig. 3:**
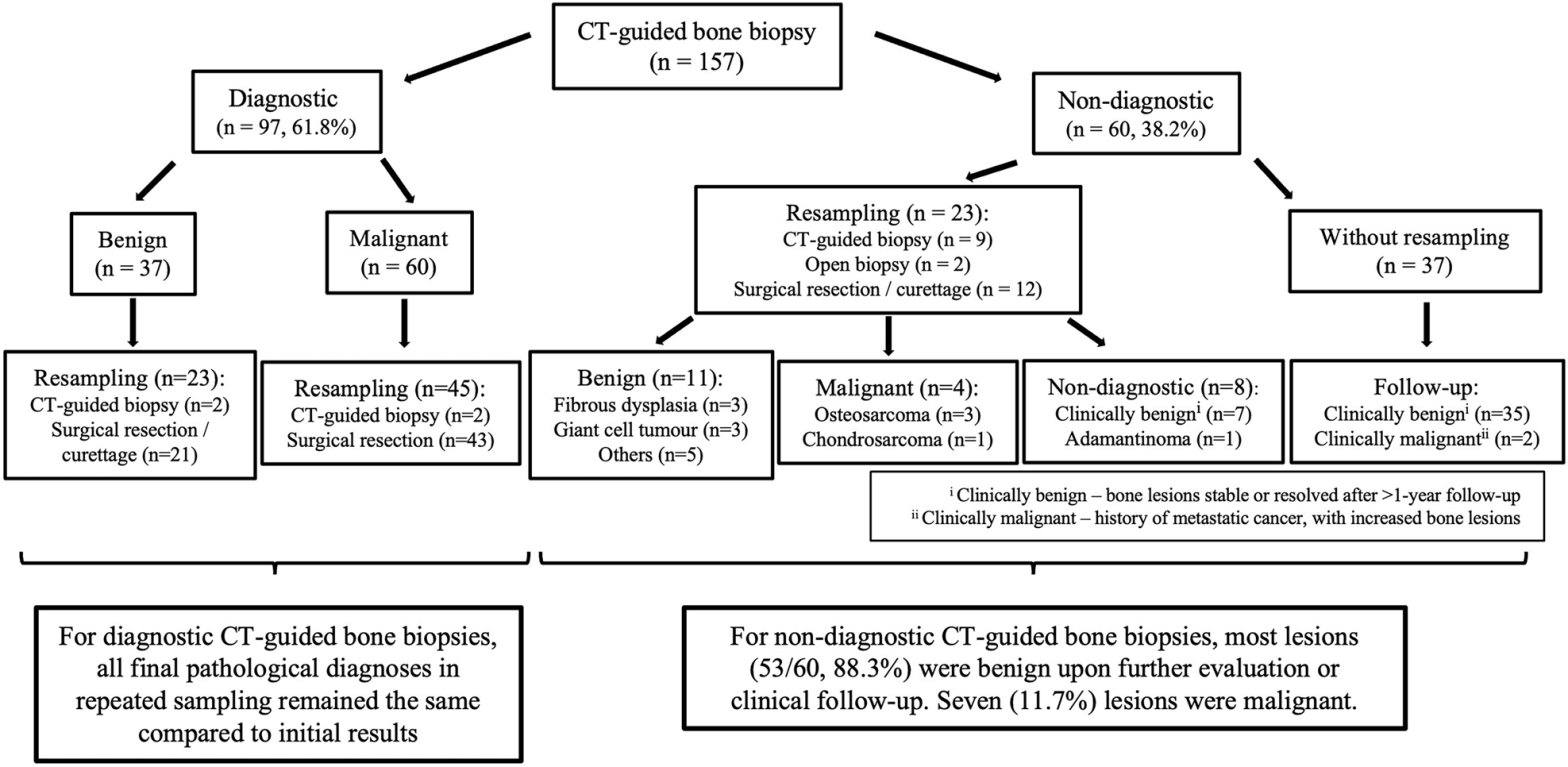
Flow diagram of the outcomes of CT-guided bone biopsies with diagnostic and non-diagnostic results

About two-thirds (61.8% [97/157]) of the CT-guided bone biopsies yielded a specific diagnosis. In these 97 lesions with diagnostic biopsy results, 60 (61.9%) were malignant lesions, and mainly osteosarcoma (*n* = 39). Biopsies with benign findings (38.1% [37/97]) were more diversified, such as aneurysmal bone cyst (*n* = 7), giant cell tumor (*n* = 7), and osteofibrous dysplasia (*n* = 6). The final pathological diagnosis was compatible with the initial CT-guided biopsy result in all cases that underwent further resection or tissue sampling (Fig. 3).

### CT-guided bone biopsies with non-diagnostic results

About one-third (38.2% [60/157]) of CT-guided bone biopsies were non-diagnostic. For these lesions, more than one-third (38.3% [23/60]) had further tissue sampling by means of repeat CT-guided biopsy (*n* = 9), open biopsy (*n* = 2), surgical resection (*n* = 2), or curettage (*n* = 10). For the nine lesions that underwent repeat CT-guided biopsy, the diagnostic rate was 44.4% (4/9), yielding osteosarcoma (*n* = 2), fibrous dysplasia (*n* = 1), and aneurysmal bone cyst (*n* = 1). When the additional diagnostic yield from repeat CT-guided biopsy was considered, the overall non-diagnostic rate of CT-guided biopsy decreased to 35.6% (56/157).

**Fig. 4:**
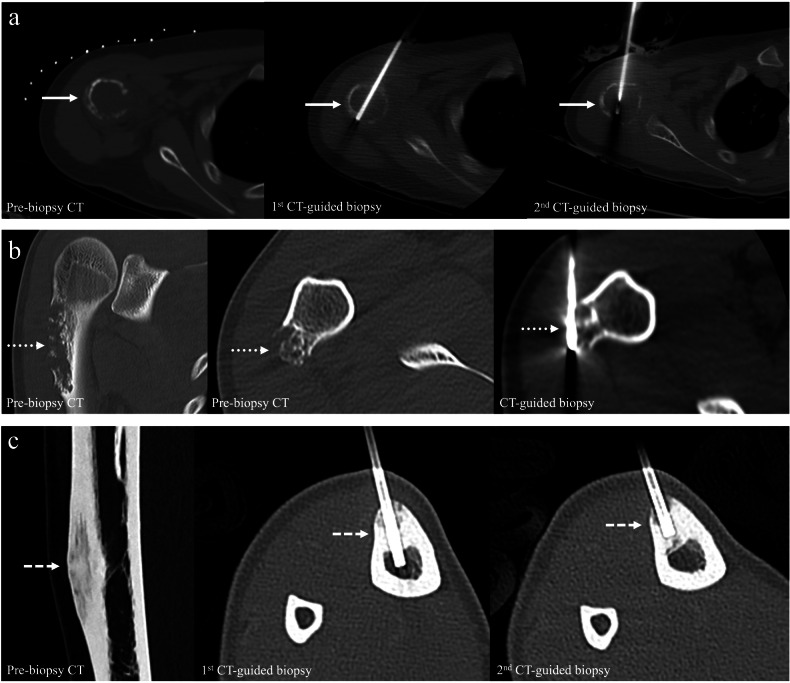
Examples of malignant bone lesions with non-diagnostic CT biopsy. **a** Initial CT-guided biopsy of a humoral lytic lesion (solid arrows) with aggressive features, including a wide zone of transition, cortical destruction, and spiculated periosteal reaction, was non-diagnostic. Osteosarcoma was confirmed on repeat CT-guided biopsy. **b** CT-guided biopsy of a lytic humeral lesion (dotted arrows) with chondroid matrix, cortical destruction, and extra-osseous soft tissue mass yielded a cartilaginous tumor without a specific diagnosis. Surgical resection was performed in view of its aggressive radiological appearance, and the final diagnosis was chondrosarcoma. **c** Initial and repeated CT-guided biopsy of the anterior tibial mixed lytic and sclerotic lesion (dashed arrows) were non-diagnostic. Histology following surgical resection revealed adamantinoma

**Fig. 5:**
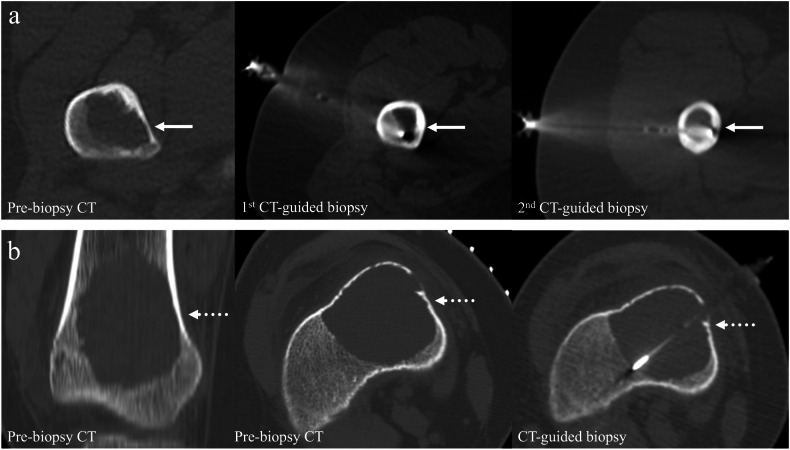
Examples of benign bone lesions with non-diagnostic CT biopsy. **a** Initial CT-guided biopsy of the proximal femoral lytic lesion (solid arrows) was non-diagnostic. Fibrous dysplasia was confirmed in the second CT-guided biopsy. **b** CT-guided biopsy of a distal femoral lytic lesion involving the epiphysis (dotted arrows) was non-diagnostic. Curettage was subsequently performed, and a giant cell tumor was confirmed pathologically

Five malignant bone lesions were confirmed on the second tissue sampling. Two cases of osteosarcoma were confirmed on repeat CT-guided biopsy. One case of osteosarcoma and one case of chondrosarcoma were revealed on pathological examination of resected surgical specimens. One further patient underwent CT-guided bone biopsy twice with non-diagnostic results, though the pathology of the resected bone lesion revealed malignant adamantinoma. Examples are shown in Figs. 4 and 5.

**Fig. 6:**
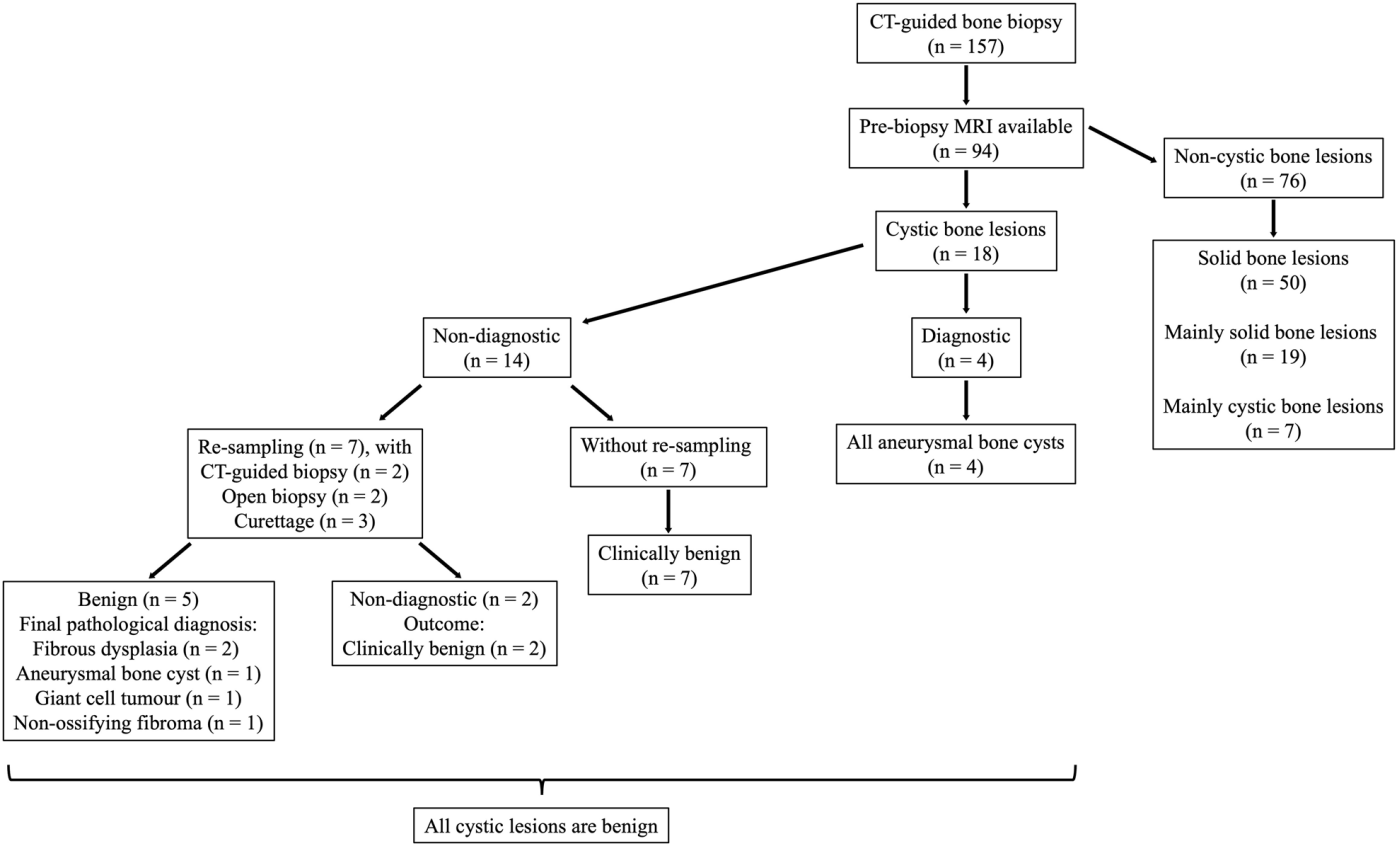
Flow diagram of the outcomes of CT-guided biopsies of cystic bone lesions

Eleven benign bone lesions were confirmed in the second tissue sampling, with diverse pathologies, including fibrous dysplasia (*n* = 3) and giant cell tumor (*n* = 3) (Fig. 6). About one-third (34.8% [8/23]) of lesions remained non-diagnostic with a second pathological examination. All patients who had repeated non-diagnostic biopsy results had complete resolution of symptoms, with the bone lesion either static or smaller in size after follow-up of more than one year. These lesions were considered clinically benign.

Most patients (61.7% [37/60]) with non-diagnostic CT-guided bone biopsy did not undergo further tissue sampling. Of these cases, most (94.6% [35/37]) were considered clinically benign, with symptom resolution, and static or smaller bone lesions on follow-up imaging. Two patients, one with metastatic breast cancer diagnosed at age 17 years, and the other with metastatic undifferentiated sarcoma diagnosed at age 11 years, did not undergo further tissue sampling, as their bone lesions demonstrated interval progression in size and number and were deemed clinically malignant (Fig. 3).

### Clinical and radiological characteristics of bone lesions with non-diagnostic versus diagnostic CT-guided biopsies

Patients who presented with symptomatic bone lesions were more likely to have a diagnostic biopsy result (*p* = 0.03). Patient age, sex, history of malignancy, type or size of biopsy needle, including use of forceps, were not related to the likelihood of obtaining a diagnostic biopsy result., However, biopsies with fewer passes (*p* = 0.02) and shorter (*p* = 0.01) tissue cores tended to yield non-diagnostic results. Bone lesions with non-diagnostic CT-guided biopsy results were smaller (*p* = 0.004), more often purely lytic (*p* = 0.049), and less likely mixed lytic and sclerotic (*p* = 0.004) than bone lesions with diagnostic results. There were also significant differences in other imaging features between the two groups. Non-diagnostic lesions were more likely to have narrow transition zones (*p* < 0.001) and sclerotic margins (*p* < 0.001) and less likely to have cortical destruction (*p* < 0.001), periosteal reaction (*p* < 0.001), or an extra-osseous soft tissue mass (*p* < 0.001). Comparison of clinical and radiological characteristics of bone lesions with non-diagnostic versus diagnostic CT-guided biopsies is shown in Table 4.

**Table 4:** Clinical and radiological characteristics of bone lesions with non-diagnostic versus diagnostic CT-guided biopsies

Parameter	Non-diagnostic CT-guided bone biopsies (*n* = 60)	Diagnostic CT-guided bone biopsies (*n* = 97)	*p*-value
Age (years), mean ± SD	13.2 ± 5.0	14.4 ± 4.2	0.26
Male sex, no. (%)	31 (51.7)	64 (66.0)	0.09
Known malignancy, no (%)	5 (8.3)	9 (9.3)	1.00
Symptomatic, no (%)	51 (83.3)	93 (95.9)	**0.03**
Biopsy system/needle size			
Outer co-axial (gauge), median (range)	11 (9–16)	13.5 (8–17)	0.49
Inner biopsy needle (gauge), median (range)	16 (13–18)	16 (13–18)	0.56
No. of biopsy passes, median (range)	3 (1–8)	4 (1–11)	0.33
No. of tissue cores obtained, median (range)	1 (1–7)	3 (1–10)	**0.02**
Length of tissue cores (cm), mean ± SD	1.1 ± 1.3	1.4 ± 0.9	**0.01**
Additional biopsy with forceps, no. (%)	11 (18.3)	14 (14.4)	0.51
Lesion nature in MRI			**0.002**
Solid, no. (%)	15/43 (34.9)	35/51 (68.6)	**0.002**
Predominantly solid, no. (%)	11/43 (25.6)	8/51 (15.7)	0.30
Predominantly cystic, no. (%)	3/43 (7.0)	4/51 (7.8)	1.00
Cystic, no. (%)	14/43 (32.6)	4/51 (7.8)	**0.003**
Monostotic, no. (%)	52 (86.7%)	80 (82.5)	0.65
Maximum lesion diameter, mean ± SD	5.1 ± 3.6	6.8 ± 4.2	**0.004**
Density in CT			**0.002**
Lytic, no. (%)	38 (63.3)	45 (46.4)	**0.049**
Sclerotic, no. (%)	6 (10.0)	3 (3.1)	0.09
Mixed lytic and sclerotic, no. (%)	16 (26.7)	49 (50.5)	**0.004**
Narrow transition zone, no. (%)	49 (81.7)	32 (33.0)	**< 0.001**
Sclerotic margin, no. (%)	28 (46.7)	17 (17.5)	**< 0.001**
Cortical destruction, no. (%)	8 (13.3)	61 (62.9)	**< 0.001**
Periosteal reaction, no. (%)	9 (15.0)	42 (43.3)	**< 0.001**
Extra-osseous soft tissue mass, no. (%)	4 (6.7)	48 (49.5)	**< 0.001**

Figures in bold indicate statistical significance with *p* < 0.05

*SD* standard deviation, *no.* number

### Cystic bone lesions

Ninety-four (59.9%) of the 157 lesions with CT-guided biopsy had pre-biopsy MRI available for assessment. The mean interval between MRI and subsequent biopsy was 36.4 ± 54.1 days. About half (53.2% [50/94]) of 94 bone lesions were solid, with the remainder being predominantly solid (20.2% [19/94]), predominantly cystic (7.4% [7/94]), and cystic (19.1% [18/94]) lesions. All cystic bone lesions were either pathologically proven to be benign or clinically considered benign after follow-up (Fig. 6). There were significantly more solid lesions in the diagnostic biopsy group (*p* = 0.002), and conversely, significantly more cystic lesions in the non-diagnostic group (*p* = 0.003). No significant difference was observed between the two groups with respect to the proportion of predominantly solid or predominantly cystic lesions (Table 4).

### Pre-procedural CT assessment

For non-diagnostic bone lesions, 88.3% (53/60) were considered non-aggressive on pre-procedural CT, and were all ultimately benign. Conversely, 11.7% (7/60) were considered aggressive on pre-procedural CT, and were all ultimately malignant.

### Inter-observer agreement of MRI and CT assessment of bone lesions

There was substantial to almost perfect agreement in classifying bone lesions on MRI according to their proportion of solid and cystic components (κ = 0.87, 95% CI: 0.78–0.95). This was also seen when evaluating the radiological features of the bone lesions on CT, including matrix density (κ = 0.92, 95% CI: 0.86–0.98), zone of transition (κ = 0.95, 95% CI: 0.90–1.00), as well as presence of a sclerotic margin (κ = 0.95, 95% CI: 0.90–1.00), cortical destruction (κ = 0.94, 95% CI: 0.88–0.99), periosteal reaction (κ = 0.92, 95% CI: 0.85–0.98), and an extra-osseous soft tissue mass (κ = 0.92, 95% CI: 0.85–0.98) (Table 5).

**Table 5:** Inter-observer agreement of MRI and CT assessment of bone lesions

Parameter	Cohen’s kappa coefficient (κ)	Agreement
Lesion nature in MRI (solid, predominantly solid, predominantly cystic, cystic)	0.87 (95% CI: 0.78–0.95)	Almost perfect
Density in CT (lytic, sclerotic, and mixed lytic and sclerotic)	0.92 (95% CI: 0.86–0.98)	Almost perfect
Narrow transition zone	0.95 (95% CI: 0.90–1.00)	Almost perfect
Sclerotic margin	0.95 (95% CI: 0.90–1.00)	Almost perfect
Cortical destruction	0.94 (95% CI: 0.88–0.99)	Almost perfect
Periosteal reaction	0.92 (95% CI: 0.85–0.98)	Almost perfect
Extra-osseous soft tissue mass	0.92 (95% CI: 0.85–0.98)	Almost perfect

*CI* confidence interval

## Discussion

In this study, over one-third (38.2%) of 157 CT-guided bone biopsies in pediatric patients yielded non-diagnostic results at the first attempt. This was comparable to a previous study performed in a pediatric population, which had a 32.6% non-diagnostic rate in 89 CT-guided bone biopsies [14]. A study of 800 adult CT-guided biopsies also had a similar non-diagnostic rate of 31.1% [10]. Factors associated with non-diagnostic CT-guided biopsy included incidental bone lesions, smaller lesion size, and purely lytic bone lesions. Also, bone lesions with a narrow zone of transition, a sclerotic margin, as well as an absence of cortical destruction, periosteal reaction, or extra-osseous soft tissue were more likely to yield non-diagnostic results. These characteristics are usually seen with non-aggressive bone lesions [1, 2]. Although CT-guided bone biopsy in this study of pediatric patients produced considerable non-diagnostic results, a substantial proportion of these lesions were ultimately benign. This contrasted with CT-guided bone biopsies in middle-aged and older adults, where nearly half of non-diagnostic lesions were subsequently pathologically confirmed to be malignant [9, 10]. As the decision to proceed with biopsy is typically made through multidisciplinary tumor board discussions involving pediatric orthopedic surgeons, radiologists, pediatric oncologists, and pathologists, it is important to recognize that most non-diagnostic CT-guided bone biopsies in pediatric patients ultimately correspond to benign lesions. As most of these bone lesions were symptomatic, patients and their families would be understandably worried about the underlying nature of these lesions. The uncertainty of non-diagnostic pathological results after CT-guided biopsy can add to their anxiety. However, it is reassuring to note that in the presence of non-aggressive radiologic features, a non-diagnostic biopsy result is more likely to reflect a benign process. Communicating this likelihood can help mitigate anxiety and support shared decision-making, especially when considering conservative management strategies [14, 16].

However, not all bone lesions with non-diagnostic CT-guided biopsy results were benign. Contrary to the previous study by Kasalak et al, which found no malignant lesion in repeated sampling and follow-up of non-diagnostic lesions [14], seven cases in this study were eventually pathologically proven or clinically considered to be malignant. Amongst these cases, there were three osteosarcomas. Their radiological features were consistent with an aggressive lesion, with a wide zone of transition, cortical destruction, spiculated periosteal reaction, and extra-osseous soft tissue mass [18]. Repeated CT-guided biopsies were performed at different segments of these osteosarcoma cases and yielded diagnostic results for each of them. The initial non-diagnostic result could be due to sampling of necrotic tumoral areas. There was one case of chondrosarcoma, in which the initial CT-guided biopsy yielded a cartilaginous tumor without a specific diagnosis. Surgical resection was performed in view of its aggressive radiological appearance, including cortical destruction and extra-osseous soft tissue mass. Core biopsy for cartilaginous tumors is prone to sampling error due to tissue heterogeneity, which might explain why the initial biopsy was non-diagnostic [19–21]. There was also one case of a mixed lytic and sclerotic lesion of the anterior tibial cortex with cortical destruction, which yielded non-diagnostic results on initial and repeated CT-guided biopsy. The lesion was subsequently resected and confirmed to be an adamantinoma. Osteofibrous dysplasia and adamantinoma are considered parts of the same disease spectrum, and differentiation is challenging both radiologically and pathologically [22, 23]. The final two aggressive lesions with non-diagnostic results occurred in two patients with known metastatic breast cancer and metastatic sarcoma, respectively, both presented with sclerotic bone lesions. Although CT-guided bone biopsies were non-diagnostic, the lesions were clinically deemed malignant as they progressed in size and number. No further tissue sampling was performed as the clinical condition of the patients deteriorated. The diagnostic yield of sclerotic lesions is lower than that of lytic non-cystic lesions. This is possibly due to bone sclerosis occasionally being reactive rather than tumoral in nature, as well as technical difficulty in obtaining a tissue sample from dense sclerotic bone, which may lead to a crushed tissue sample [11, 24].

Pre-biopsy MRI examination enables lesions to be classified according to their cystic and/or solid proportions [25]. Cystic bone lesions were more likely to yield a non-diagnostic result, though all were ultimately benign lesions. For these lesions, clinical and radiological follow-up may be more beneficial than biopsy, which has a low diagnostic yield. When cystic or predominantly cystic bone lesions are encountered during CT-guided biopsy, emphasis should be placed on targeting any solid components, no matter how small [26, 27]. Biopsy samples can also be obtained along the edge of the lesion encompassing some peritumoral bone [26]. Other techniques to increase the diagnostic yield of CT-guided bone biopsy include the additional use of forceps. Flyer or endomyocardial biopsy forceps have been reported in the literature to increase the size of the tissue sample obtained, reducing non-diagnostic results due to sample fragmentation [28, 29]. However, this was not observed in our study. Aspiration of fluid or blood clot during CT-guided biopsy of cystic bone lesions for cytological analysis can also be considered. Cytological assessment of the aspirated blood can help establish a diagnosis, though fluid aspirate may not necessarily be achievable in all cystic bone lesions [26, 30]. The current study conforms with previous findings that increasing the number (> 3 tissue cores) and length (≥ 1 cm) of tissue cores yielded increased diagnostic yield [27].

Multidisciplinary team involvement, including orthopedic surgeons, radiologists, pathologists, and oncologists, is crucial in clinical decision-making for suspected primary bone tumors in children. Radiological surveillance is preferred for “do-not-touch” asymptomatic lesions with classic imaging features. When the diagnosis is not clear based on imaging appearances, clinical symptoms, or interval lesion growth, biopsy is usually indicated to delineate the nature of the lesion and exclude malignancy. The current study reaffirms that if pre-biopsy imaging features are non-aggressive and biopsy is non-diagnostic, regular surveillance is a reasonable management option [14]. Conversely, when imaging appearances are aggressive, repeat bone biopsy, with multidisciplinary team input, is strongly recommended [5, 10]. In addition to assessing the cellular and tissue morphology of the biopsy sample, pathologists frequently correlate these morphological features with clinical and radiological findings. They then often undertake various ancillary analytic tests, which may include immunohistochemical staining, cytogenetics, and molecular analysis, to arrive at a pathological diagnosis [31].

There are several limitations of this study. Firstly, CT-guided bone biopsy techniques were not standardized, reflecting the multi-institutional nature of this study. Secondly, different radiologists and pathologists with varying experiences from three different institutions performed the biopsy procedures and analyzed the histological samples. This does, however, reflect usual clinical practice. Third, some of the MRI examinations at diagnosis were performed at outside institutions prior to referral, and retrospective review of these studies was not feasible, thereby limiting the number of cases with MRI available for analysis. Given the 10-year study period, the adoption of advanced MRI sequences, such as diffusion-weighted imaging and dynamic contrast-enhanced sequences, was inconsistent and varied across the three participating centers. To ensure uniformity and reflect a pragmatic clinical scenario, we limited our analysis to three core sequences: unenhanced T1W, fat-suppressed T2W, and post-contrast T1W images, which were consistently obtained in all centers throughout the study period. Fourth, in a small portion of lesions where biopsies were obtained prior to 2016, where the availability of molecular genetic testing can be limited, further subtyping of the sarcoma diagnoses was not possible based on morphology and immunohistochemistry, and therefore concluded to be high-grade sarcoma. Finally, the current study primarily considered the non-diagnostic rate of the first CT-guided biopsy attempt. While repeating CT-guided biopsy may help increase the overall diagnostic rate, such tissue sampling is not without risk, especially the need for repeated general anesthesia, potential risk of neurovascular injury, and exposure to ionizing radiation in this young population. We believe this approach reflects real-world clinical practice, where the decision to pursue repeat tissue sampling versus observation is guided by a careful evaluation of the individualized risk–benefit ratio.

In conclusion, approximately one-third of CT-guided bone biopsies in pediatric patients yielded non-diagnostic pathological results. For lesions with aggressive imaging features on CT, repeat tissue sampling—either via repeat percutaneous or open biopsy—is warranted, as all such cases were ultimately malignant in our cohort. All lesions with non-diagnostic histopathological results following CT-guided bone biopsy and non-aggressive imaging features were subsequently found to be benign. Factors associated with non-diagnostic CT-guided biopsy results included incidental bone lesions, smaller lesion size, cystic nature on MR, small core numbers, and shorter tissue cores. Additional CT features such as a narrow zone of transition, sclerotic margins, and absence of cortical destruction, periosteal reaction, or extra-osseous soft tissue were also associated with non-diagnostic outcomes. In the setting of multidisciplinary care for patients with suspected primary bone tumors, CT-guided bone biopsy with non-diagnostic histopathological results strongly favors benignity in lesions with non-aggressive imaging features and should guide management towards a more conservative approach.
